# Coupled VO_2_ Oscillators Circuit as Analog First Layer Filter in Convolutional Neural Networks

**DOI:** 10.3389/fnins.2021.628254

**Published:** 2021-02-11

**Authors:** Elisabetta Corti, Joaquin Antonio Cornejo Jimenez, Kham M. Niang, John Robertson, Kirsten E. Moselund, Bernd Gotsmann, Adrian M. Ionescu, Siegfried Karg

**Affiliations:** ^1^IBM Research Zürich, Rüschlikon, Switzerland; ^2^Department of Engineering, University of Cambridge, Cambridge, United Kingdom; ^3^Nanoelectronic Devices Laboratory, École Polytechnique Fédérale de Lausanne, Lausanne, Switzerland

**Keywords:** oscillatory neural network, vanadium dioxide, phase-encoding, convolutional neural networks, pattern recognition, relaxation oscillators, coupled oscillators

## Abstract

In this work we present an in-memory computing platform based on coupled VO_2_ oscillators fabricated in a crossbar configuration on silicon. Compared to existing platforms, the crossbar configuration promises significant improvements in terms of area density and oscillation frequency. Further, the crossbar devices exhibit low variability and extended reliability, hence, enabling experiments on 4-coupled oscillator. We demonstrate the neuromorphic computing capabilities using the phase relation of the oscillators. As an application, we propose to replace digital filtering operation in a convolutional neural network with oscillating circuits. The concept is tested with a VGG13 architecture on the MNIST dataset, achieving performances of 95% in the recognition task.

## Introduction

Convolutional Neural Networks (CNNs) are the architecture of choice to compute image recognition tasks. Widely used in commercial technology for their recognition accuracy, they are hindered in speed and power efficiency by the frequent access to the memory they need to perform to train a high number of parameters for each convolutional layer in deep networks (Sebastian et al., 2020). The development of neuro-inspired hardware holds the promise of accelerating these algorithms by exploiting in-memory computing concepts and limiting the number of accesses to the memory. A system of coupled oscillator, or Oscillatory Neural Network (ONN) can be used to store and recognize multiple patterns in compact networks. As described in Hoppensteadt and Izhikevich (1999) and Izhikevich (2000), systems of coupled oscillators lock in frequency and establish programmable phase relations that can be used for in-time computing applications. An ONN comprises a system of oscillators, in the role of neurons, connected to each other with synaptic weights, that represent the strength of the oscillators’ coupling and the memory of the network. The ONN systems therefore rely on encoding and processing the information with time-delays in the circuits, rather than the amplitude of a signal, therefore being resilient to voltage noise and to scaled power supply.

**FIGURE 1:**
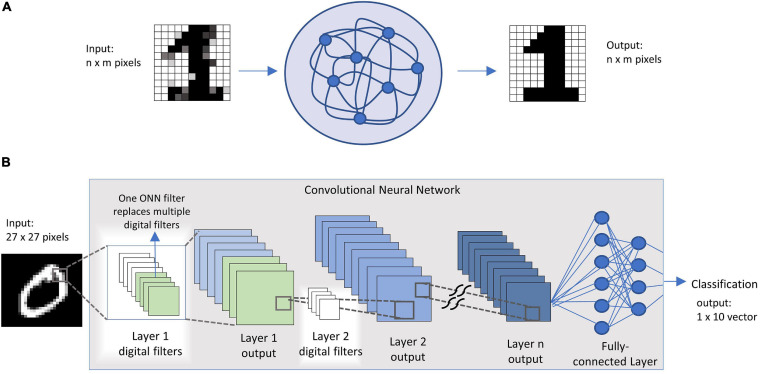
**(A)** image inference process in an oscillatory neural network; **(B)** image classification through a convolutional neural network.

Exploiting the associative memory capabilities of such networks, tasks as image recognition can be performed. Numerous works have simulated through mathematical and circuit simulations the digit pattern retrieval with different coupled oscillators technologies: (Jackson et al., 2015) shows 20-pixel digit recognition using transition metal oxides and resistive ram technology; (Nikonov et al., 2015; Liyanagedera et al., 2016), perform similar simulations on Spin Torque Oscillators (STOs); (Hölzel and Krischer, 2011) with Van der Pool oscillators. These works are based on storing patterns with *n × m* pixels into an ONN that comprises *n × m* oscillators. To perform the recognition, a distorted pattern of the same pixel size is fed to the ONN, and using the minimum phase attractor of the circuit, the right stored pattern is retrieved. The output is an *n × m* pixels image of the correct digit. Image classifications tasks, however, work quite differently. Taking as an example digit classification through a neural network, an image of *n × m* pixels if fed into the network. The network output is an 1 × 10 array containing the classification probabilities of that image. This classification operation is most commonly performed by convolutional neural networks, that process the image with a series of trainable convolutional filters in the first few layers and achieve recognition after some fully connected layers (Figure 1).

The link between convolutional neural networks and the associative memory capabilities of oscillatory neural networks has so far been explored, to our knowledge, only in Liu and Mukhopadhyay (2018), where an associative memory bank (Hopfield network) replaces the fully connected layers in CNNs. The associative memory is here used to perform a classification with a combination of unsupervised learning and transfer learning techniques. Even though the concept is very interesting and promising, the Hopfield network that this technique envisions comprises between 256 and 2,024 neurons. However, the physical demonstrations of oscillatory neural networks features maximum 100 oscillators as neurons with standard Phase Locked Loop or equivalent CMOS technology (Jackson et al., 2018). The technological challenge in the physical realization of large oscillatory neural networks resides in the complex dynamics of the oscillators’ frequency and phase synchronization when the electrical components are affected by variability. This is even more true when the ONNs are built with novel oscillator technology, such as STOs or vanadium-dioxide (VO_2_) oscillators (Romera et al., 2018; Raychowdhury et al., 2019), for which a maximum of 6 oscillators have been connected into a network.

Alternatively, it is suggested that the computing capabilities of small oscillator networks, with up to 10 nodes, can be efficiently exploited to do various image processing tasks, like graph coloring or image saliency processing (Cotter et al., 2014; Tsai et al., 2016). These previous works propose an ONN scheme in which the computation is based on the distance between the input image and the feature to be recognized. For example in Tsai et al. (2016) this distance is encoded in the difference in gate voltages between two transistors which bias a phase transition device. Another popular configuration encodes the distance between the input pattern and the feature to be recognized in a frequency shift between oscillators, which are connected by a fixed coupling. The distance between the two patterns is then calculated on the time the oscillators need to converge to the same frequency (Cotter et al., 2014; Nikonov et al., 2015; Zhang et al., 2019). The implementation of these concepts does not use the associative memory capabilities of the ONNs to store multiple patterns. Instead, to perform the distance measure, the circuit needs to be reconfigured each time a different feature needs to be recognized. In our work, we propose an implementation of small, fully connected networks, which exploit the associative memory capabilities of an Hopfield network. This allows to store the different features to be recognized in the same network, and enables the recognition of up to 5 different features within one computation performed by the same filter. In addition, in our work we provide the missing the link to show how the feature extraction performed by ONNs can be used for image classification tasks. We show the potentiality of the ONN technology for the realization of reconfigurable CNN in hardware, therefore bridging the gap between previous demonstration of ONN pattern retrieval and the industry-standard algorithms.

Among the new oscillator technologies, we concentrate our analysis on VO_2_ oscillators, as they offer the advantage of realization of very compact oscillators, which can be easily coupled with standard electrical components, allowing for easy reconfigurability of the system (Parihar et al., 2015; Corti et al., 2018). VO_2_ based oscillators also offer good scalability perspective and demonstrate operating voltage of less than 1 V and low power consumption (∼20 μW per oscillator) (Shukla et al., 2016).

We exploit the feature extraction capabilities of small networks of VO_2_ coupled oscillator to replace digital filters in CNNs (Figure 1B). We fabricate VO_2_ oscillators on a Si platform, adopting a crossbar (CB) configuration with scaled device dimensions down to 70 nm. The CB devices exhibit improved variability and reliability compared to co-planar structures and enable the coupling of 4 oscillators. We demonstrate that such a 4-node ONN can memorize and perform 5 different filtering actions of a CNN in a single circuit. Simulations with a 3 × 3 ONN further show how the concept can be applied to replace digital filters in the first layer of a CNN with a VGG-13 inspired architecture and through the adoption of a transfer learning technique. The hybrid CNN-ONN platform has been tested on the MNIST algorithm reaching recognition performances up to 95%. As an outlook, we discuss the benchmark of this technology when extended to all the layers of a CNN, up to the fully connected layers, in comparison with existing hardware and conclude that ONNs might be used as fast and low-power inference machines.

## Materials and Methods

### Device Fabrication

VO_2_ is a phase change material that presents a volatile, temperature driven insulator to metal transition (IMT). The transition can be triggered by joule heating when a voltage is applied to a VO_2_ device (Kim et al., 2010), and it is reversed when the voltage across the device is removed. Given its volatile phase-change characteristics, VO_2_ cannot be used as memory element like chalcogenide-based phase change materials (PCM). However, the VO_2_ phase transition can be instead exploited to build very compact oscillators. Other materials have shown similar properties, for example tantalum oxide (Jackson et al., 2015; Sharma et al., 2015) or niobium oxide (Li et al., 2015), however, the near-room temperature phase transition of VO_2_ and its proven high endurance up to 10^9^ cycles (Shukla et al., 2015) make this material a most favorable choice of oscillators-based technology. VO_2_ can be grown crystalline on TiO_2_ substrates; however, when deposited on Si, the film forms grains of the average dimension of ∼50 nm (Premkumar et al., 2012). In the interest of future integration with electronics we have chosen to focus on integration on silicon in our work.

**FIGURE 2:**
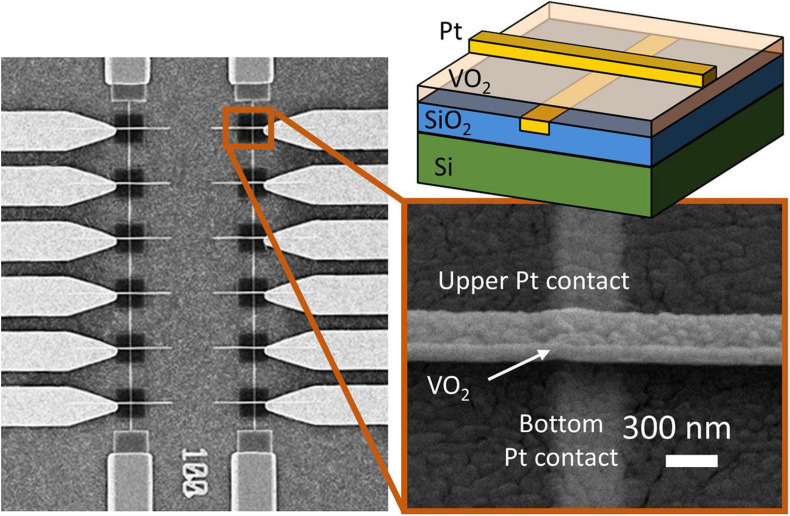
Left: scanning electron microscopy (SEM) image of 12 VO_2_ devices. Top right: schematic of a VO_2_ device deposited on a Si/SiO_2_ substrate. The device area is defined by the width of the Pt contact lines. On the bottom, a SEM image of a 350 nm device. Minimum device dimension demonstrated: 70 nm.

**FIGURE 3:**
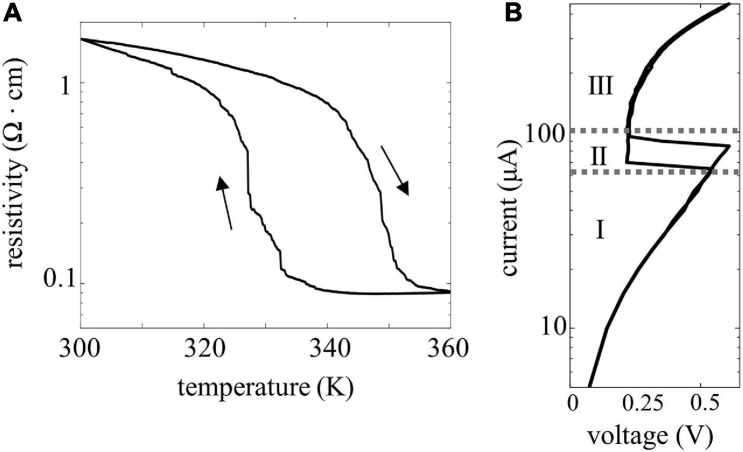
**(A)** Resistivity measure of a 300 nm × 300 nm crossbar VO_2_ device. The insulator to metal phase transition happens at around 340 K and registers 2 orders of magnitude phase change. **(B)** IV curve of a VO_2_ device. Three different areas can be identified: the insulating region (I), the negative differential resistance region (II), and the metallic region (III).

Figure 2 shows VO_2_ devices fabricated in a CB geometry on a 4” Si wafer with a 1 μm thermal SiO_2_ layer. Trenches are etched into the SiO_2_ substrate and filled with Pt to provide the bottom electrode. Subsequently, an 80 nm thick VO_2_ film is grown via atomic layer deposition and post-annealed, resulting in a policrystalline, granular film (Bai et al., 2020; Niang et al., 2020). Finally, top electrodes are formed using e-beam lithography and Pt evaporation. The smallest device area is 70 nm × 70 nm allowing a very compact design. The resistivity vs. temperature curve (RT) of a 250 nm × 250 nm device is shown in Figure 3A and exhibits an insulator-to-metal phase-transition with roughly a two-order of magnitude in resistance change. The step-like RT implies multi-grain transitions, as already shown in previous work (Ruzmetov et al., 2009; Takami et al., 2014; Corti et al., 2019). Figure 3B shows the insulator-to-metal and metal-to-insulator transition of an electrically activated device. A current source is used to control the current flowing in the device; a voltmeter is used to measure the voltage at each point. The IV characteristic of this device shows three different operating regions: a first region, in which the device is in its high resistance state, a negative differential resistance regime upon the phase change, and finally the low resistance region. A crossbar-geometry of VO_2_ based-oscillators applications has previously been fabricated with point-probe contacts on TiN on Si, yielding record-speed oscillations performances of 9 MHz, almost an order of magnitude more than what demonstrated with planar structures (Mian et al., 2015). In another more recent work, the oscillating and coupling dynamics of such a vertical structure have been measured and modeled (Tobe et al., 2020). In this work, we further report that the cross-bar geometry yields a better reliability of the devices. In fact, in a previous work we discussed how coplanar devices needed a burn-in cycle to initialize the devices, which sometimes resulted in fatal irreversible changes in morphology (Corti et al., 2019). The crossbar devices do not need a burn-in cycle, improving reliability as virtually all the devices fabricated were able to produce oscillations. Compared to the planar VO_2_ structure, the crossbar structure provides improved threshold voltage stability (device-to-device variability lowered from 20% to 10%) and resistance variability (from 10% to ∼5%). Compared to other demonstrations on silicon, the improved variability allows for coupling of more oscillator nodes, up to 4. However, to go to larger networks, careful material and device development is necessary to bring this figure down. The devices are tested in temperature-controlled chamber at 320 K and connected in the circuit configurations through external electrical components.

### Oscillatory Neural Network

**FIGURE 4:**
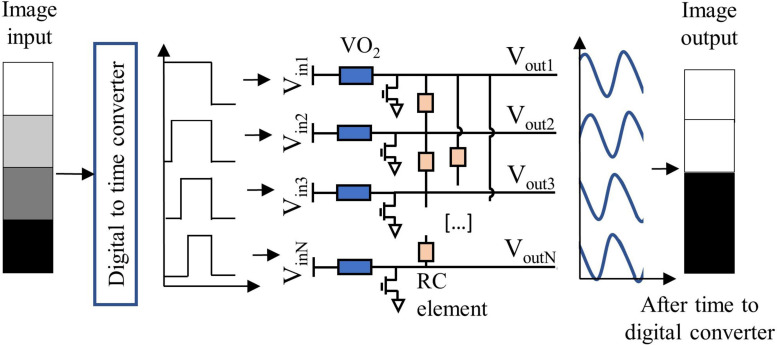
The coupled oscillator network serves as an image filter. The image input is converted into a delay of the oscillators’ input signal. A single oscillator unit comprises a VO_2_ phase-change element in series with a transistor. The coupling is realized with an externally connected resistance and a capacitance. The capacitance value is fixed, while the resistance value can be changed to store different patterns in the network and can be later substituted with a memristor.

**FIGURE 5:**
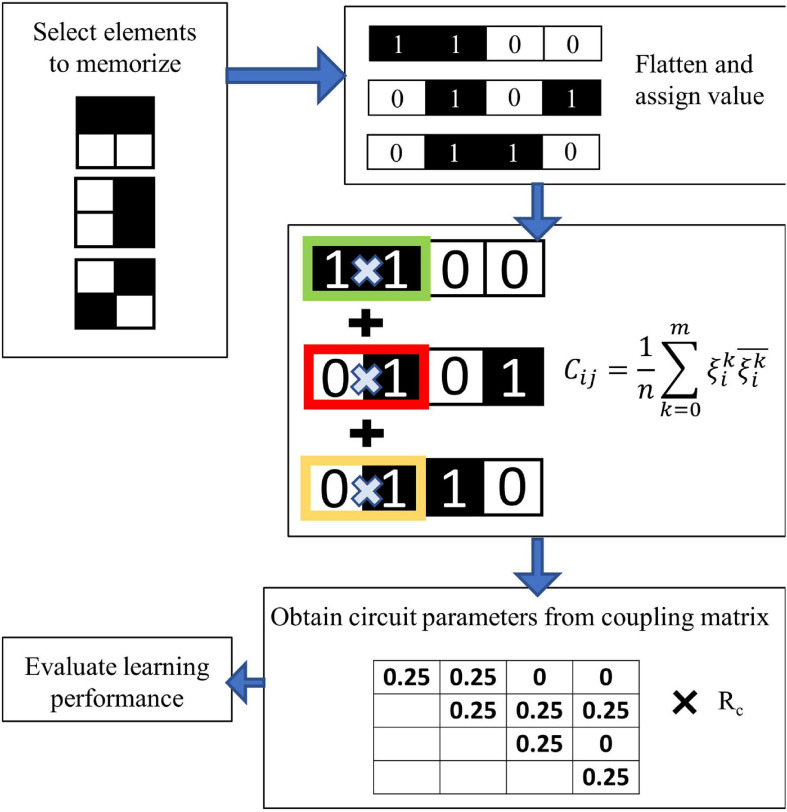
Flow chart of the learning. Weights are assigned to the pixel of each training image; the Hebbian Learning Rule is used to compute the coupling weights, which are translated into circuit values of the coupling resistance R_*c.*_

A single oscillator unit is realized biasing the VO_2_ device in the negative differential resistance regime with a series transistor as described in Parihar et al. (2014). When the device is in its insulating state the bias voltage drops mainly across the VO_2_, until the Joule heating brings its local temperature above the phase transition, and the device switches to its metallic state. When the device is in the metallic state, the voltage drops mainly across the series transistor. When the bias is chosen such that the voltage drop does not exceed the upper threshold of the negative-differential resistance regime, Joule heating is reduced. The VO_2_ device therefore cools and eventually switches back to its insulating state. The switching between the insulating and metallic state is therefore continuous and self-sustained, originating relaxation oscillations at the drain voltage of the transistor. The oscillators are coupled via resistive and capacitive elements, as shown in Figure 4, which ensure frequency and phase-locking of the drain voltage signals. The strength of the coupling element *C*_*ij*_ that connects oscillator *i* with oscillator *j* can be calculated starting from the patterns to be memorized, via the Hebbian Learning Rule (HLR) (Hoppensteadt and Izhikevich, 1999):

$$C_{i\,j}=\frac{1}{n}\,\sum_{k=0}^{m}\vartheta_{i}^{k}\,\bar{\vartheta_{j}^{k}}$$

where *n* is the total number of pixels per each image, or equivalently the number of oscillators in the network, *ϑ_*i*_^*k*^* is the value associated to the pixel *i* of pattern *k*, and *m* is the total number of patterns to be memorized in the ONN (Figure 5). These values *C*_*ij*_ are then translated in different values of the coupling resistance R_*c*_ between the oscillators. The memorized patterns appear in the operating ONN as stable phase relations between each oscillator *i* and *j*. An oscillator in phase with the reference oscillator is translated into a white pixel; an oscillator with 180° phase difference with the reference corresponds to a black pixel. Given *m* patterns memorized in the oscillatory neural network, the oscillators can stabilize their phase only according to one of the *m* memorized patterns. When the oscillators are initialized to an unstable phase relation, they will relax to the nearest stable ensemble of phase relations, i.e., to the nearest memorized pattern. In this way, from a distorted pattern a memorized pattern is retrieved. In our system in Figure 4, the oscillators are initialized to have different phase relations via a delay of the bias voltages to each oscillator. For instance, an oscillator representing a white pixel input is switched on at a time t_*d*_ = 0 compared to a reference signal; an oscillator representing a black pixel input is switched on at a time t_*d*_ = T/2 compared to the reference signal, with T indicating the period of one oscillation. Gray-scale values correspond to proportional delays. The output is represented by the phase of the oscillating transistor drain voltage, compared to a reference. When the network is initialized in this fashion to a phase relation between the oscillators that is different from the stored, stable phase relations, the system relaxes to the nearest stable phase relation, therefore achieving recognition of a pattern. The success of the input time-delay technique for image recognition is explained in detail in Corti et al. (2020). The settling time to the desired output typically varies between 4 and 5 oscillation cycles, nevertheless, after 5 oscillation cycles the phase information becomes stable and can be read-out. The information about the recognized feature is contained solely in the relative phase of the oscillators. In the experiments presented in this paper, the input delay signal is generated through a signal generator unit (National Instruments), the output of the oscillators is acquired by a signal acquisition set-up and the phase calculated with post-processing. The circuit coupling elements are realized with external electrical resistances. As an outlook, the input time-delay can be implemented in hardware via ring oscillators, and the phase-to-digital conversion can be tackled as described in Staszewski et al. (2006). Also, the coupling resistance can be implemented with reconfigurable phase change memories (Boybat et al., 2018).

The simulations for the circuit implementation of the ONNs have been done using a Spice simulator. The VO_2_ device was simulated with a behavioral model as described in Maffezzoni et al. (2015). TensorFlow^TM^ was used for the CNN and the hybrid ONN-CNN algorithms. The TensorFlow^TM^ code is used to calculate the input delay of the driving voltage of the oscillators, as described above, from an input image, taken from the MNIST dataset. The code then launches the circuit simulation of the ONN, which are conducted in SPICE. The output of the simulation, corresponding to the pattern retrieval computed by the simulated ONN circuit, is then fed back to the TensorFlow^TM^ algorithm as an output image. The image is then processed in the subsequent CNN layers with the TensorFlow^TM^ code.

The choice of the MNIST dataset to perform this computation is justified by the reduced dimensions of the dataset itself. To obtain precise results with the simulation, a high time resolution is required, with very small time-steps for each computation. The simulations of the ONN are therefore rather slow and require an extended simulation time and many computational resources. This problem is not present in the circuit implementation, as the hardware realization is able to perform at frequencies in the order of MHz. We are, however, positive that a similar approach on a more complex dataset will yield the same results here discussed with the MNIST dataset.

## Results

### Four-Coupled Oscillators

**FIGURE 6:**
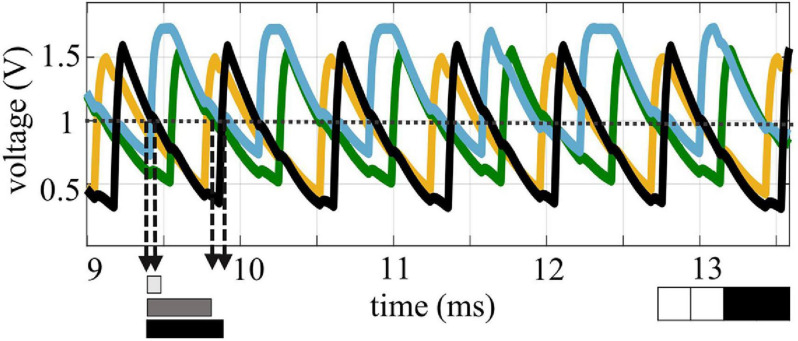
Coupling of four VO_2_ on Si oscillators. For reliable coupling, a hybrid R-C scheme was used, and the relative phase is calculated when the falling edge of the oscillations cross a 1 V threshold. In this experiment, an external capacitance of 150 nF was used on purpose to slow the oscillations, to enable a more precise sampling of the output signal.

**FIGURE 7:**
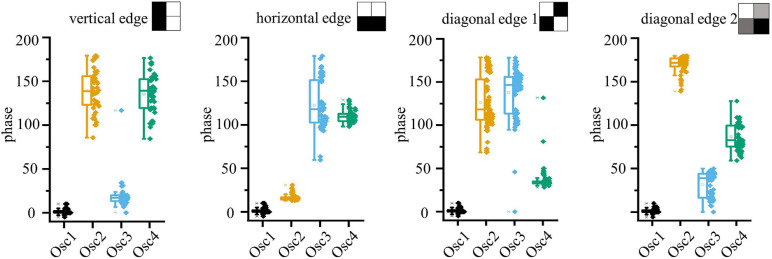
Experimental phase data that demonstrate that 4 features (e.g., vertical, horizontal and 2 diagonals) can be stored simultaneously in one 4-oscillators network. The pattern can be controlled by the time-delay of the oscillator drive. Phase-noise due to device variability impedes practical application. Circuit parameters: R_12_, R_13_, R_24_, R_34_ = 82 kΩ, R_23_, R_14_ = 130 kΩ, C_*c*_ = 5,6 nF, V_*gx*_ = 1.4 –1.6 V, V_*in*_ = 1.8–2.2 V.

In this section we present a demonstration of four coupled VO_2_ oscillators on Si, in which multiple patterns can be memorized. To form relaxation oscillator circuits, the VO_2_ resistors on a silicon wafer are coupled through externally connected resistors and capacitances. An example of the measured waveforms of four coupled oscillators is shown in Figure 6. The oscillators appear to be locked in frequency and the phase relation is calculated taking the distance between the crossing of the 1 V line in the falling edge of the oscillator curves. The coupling network has been programmed to recognize features as in a first layer of a convolutional neural networks. Looking at available analysis of feature extraction in convolutional neural networks (Zeiler and Fergus, 2014), the filters in the first layer commonly select edge features, like borders, diagonal, horizontal and vertical edges. Therefore, for the experimental demonstration, the ONN was trained to store vertical, horizontal and diagonal patterns. The weights of the circuit elements were identified through the Hebbian learning rule. To the best of our knowledge, this is the first demonstration of 4 coupled VO_2_ oscillators with memory capabilities realized on a silicon platform. The circuit parameters used for the experiments are: R_12_, R_13_, R_24_, R_34_ = 82 kΩ, R_23_, R_14_ = 130 kΩ, C_*c*_ = 5,6 nF, V_*gx*_ = 1.4–1.6 V, V_*in*_ = 1.8–2.2 V. The different values of gate voltages V_*g*_ and of the input signal V_*in*_ are used to achieve similar frequency for the oscillators, and to compensate from intrinsic differences in the devices, which present around 10% of device-to-device variability. The horizontal, vertical and diagonal patterns are identified over multiple experiments, as depicted in Figure 7. In addition, a fourth pattern in which all the oscillators result equally spaced was identified. The measurements are performed assuming Oscillator 1 as the reference oscillator; the phase of the other oscillators is calculated in respect to the crossing of the 1 V threshold of oscillator 1. Therefore, Oscillator 1 has always a phase equal to 0, with a minimal data scattering that is calculated taking into account the variability of the value of the first experimental point that crosses the 1 V line. The other oscillators present a larger scatter, which doesn’t impair the clear identification of the various patterns. However, random fluctuation of the oscillations and cross-talk noise hindered the experimental pattern recognition using the input-delay to output phase inference process. This is expected to improve with further process and design optimization of the crossbar devices.

**FIGURE 8:**
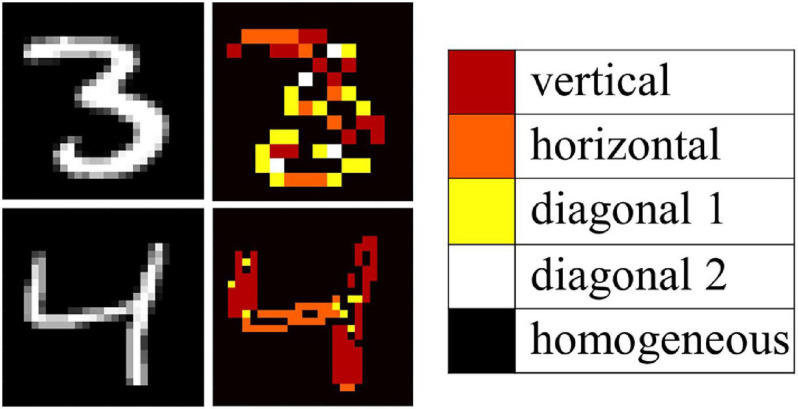
Simulation of convolution operation on MNIST images with a 2 × 2 VO_2_ oscillator filter, which corresponds to 5 digital filters of the first layer of a CNN. The simulations are calibrated with the experimental results in Figure 7.

To demonstrate the filtering capabilities of the circuit on an entire image, without suffering from the non-idealities of the experimental demonstration, circuit simulations calibrated on the experiments were conducted using Spice, reducing the variability of the VO_2_ devices from 10% to 5% and therefore increasing the recognition accuracy. In these simulations all the 4 patterns identified in the experiments were also observed; in addition, when the input delays of the circuit were chosen to be all the same, the oscillators in simulations were all oscillating in the in-phase configuration. This is an example of identification of a spurious pattern that was not encoded with the HRL. Spurious patterns arise when the memory capacity of the oscillatory neural network, that is studied to be 0.15n patterns for a n-oscillator network, is violated (Nishikawa et al., 2004; Follmann et al., 2015). Nevertheless, in such small oscillator networks the spurious patterns can be harvested as additional information. As shown in Figure 8, when using the 2 × 2 ONN filter on an image of the MNIST dataset, vertical, horizontal and diagonal edges can be identified. In addition, the background as well as the images parts that have little contrast, can be identified through the in-phase oscillating condition. This demonstrates that a single ONN filter can operate as convolutional feature edge extraction identifying 5 different features. Compared to previous work, the identification of the features does not need to proceed sequentially feature by feature, but it is done in parallel by the same filter. Moreover, the dimensionality of the filter should match the dimensionality of the input, i.e., 2D input arrays such as images are preferably processed using 2D filters. The 4 coupled oscillators system here discussed represents the minimum hardware realization to use ONNs as filters for image feature extraction.

### ONN-CNN

**FIGURE 9:**
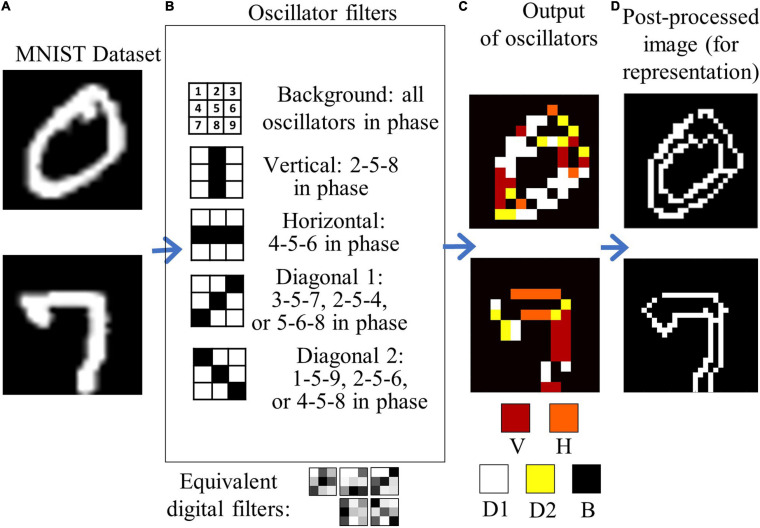
Extension of the convolution filtering operation to a 3 × 3 oscillator matrix with stride of 2. **(A)** example of handwritten digit images from the MNIST dataset. **(B)** Five digital filters are replaced with 1 ONN filter that performs equivalent edge extraction actions. The 9-bit information output is compressed in a 5-bit fashion for better representation of the edge direction in **(C)**. In **(D)**, the image in **(C)** is post-processed and expanded to a 27 × 27 pixel image to show the effectiveness of the ONN filter in recognizing the image features.

Having shown that our simulations can reproduce experimental behavior, we extend the simulations to explore the use of ONNs in combination with CNNs. The ONN circuit described in section “Oscillatory Neural Network” is simulated with Spice simulations using for the VO_2_ device a behavioral model as described in Maffezzoni et al. (2015). The simulations are done with 3 × 3 oscillators ONNs based on parameters extracted from experimental devices. A convolutional neural network with a structure similar to a VGG-13 is trained on the MNIST dataset with a standard back-propagation algorithm (Table 1). The trained weights are used to identify which features are recognized in the first layer of the CNN, that comprises 64 filters with a dimension of 3 × 3. In our network, as in Zeiler and Fergus (2014), it was also possible to identify multiple filters that selected horizontal, vertical and diagonal edges. We use the Hebbian Learning rule to store the same patterns in a 3 × 3 ONN matrix. The matrix dimension was chosen according to the dimension of the first layer convolution matrixes in the CNN. Ten thousand images from the MNIST dataset have been processed by the ONN matrix with a stride of 2, recognizing in each image vertical, diagonal, horizontal edges and uniform background. As already mentioned, storing of more than 0.15 *n* patterns, where *n* is the number of the oscillators (Follmann et al., 2015), results in the appearance of spurious patterns that can in principle hinder the feature edge extraction process. However, as already discussed for the 4-coupled oscillators experiments, the arising of spurious patterns is not detrimental for feature extraction operations. In the 3 × 3 filter case, we derived the pattern information from 3 key oscillators that oscillate in-phase for each memorized edge. For example, referring to what is depicted in Figure 9, each time oscillators 2, 5, and 8 oscillate in-phase a vertical edge is recognized, and similarly for the other edges.

**TABLE 1:** Schematic of the convolutional neural network architecture used in this work for performing the MNIST classification task.

**MNIST dataset**	**27 × 27 × 1,000**
ONN-CNN 5 ONN filters + 59 CNN filters	3 × 3 × 64, stride = 2, padding = same
CNN1	3 × 3 × 64, stride = 1, padding = same
Max pool 1	2 × 2, stride = 2, padding = same
CNN 2(×2)	3 × 3 × 128, stride = 1, padding = same
Max pool 2	2 × 2, stride = 2, padding = same
CNN 3(×2)	3 × 3 × 256, stride = 1, padding = same
Max pool 3	2 × 2, stride = 2, padding = same
Fully connected 1	4,096
Fully connected 2	1,000
Fully connected 3	10

**The network architecture is inspired by the VGG-13 architecture.**

With this technique, a dataset of 10,000 images filtered by the single ONN was calculated, with dimensions 13 × 13 × 5, where 5 represent the number of features recognized by the single ONN filter. The dataset was split in 6,000 training images and 4,000 test images.

Subsequently, five filters in the pre-trained CNN that provide the same filtered images were identified and replaced by the ONN with a simple transfer learning process:

1. The 64 CNN filters were convolved with the same images from the MNIST dataset and activated with a Relu function.
2. The CNN-filtered images were compared to the ONN-filtered images calculating the mean square error; the minimum of the mean square error was used to identify the filters from the CNN that can be substituted with the ONN.
3. A new dataset is created after the first layer, substituting the images filtered by 5 CNN filters with the 5 filtered images from the ONN.

**FIGURE 10:**
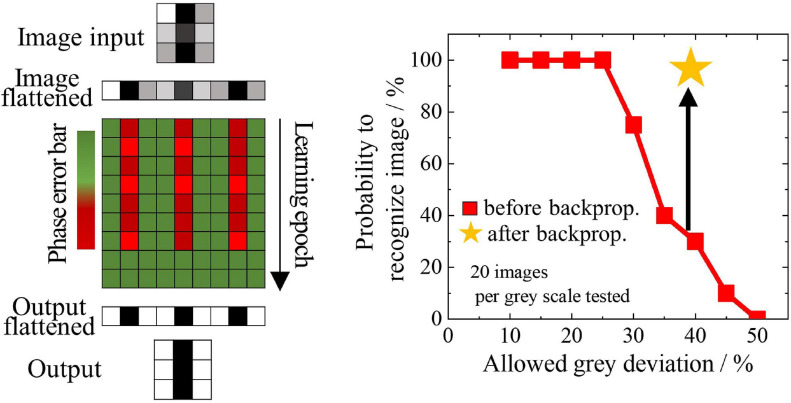
Example of backpropagation algorithm applied to a vertical edge recognition problem of the VO_2_ ONN filter. Left: a distorted edge is given as an input to the ONN filter. The difference between the expected output phase and the output phase of the filter is depicted as pixel coloring from green (no phase error) to red (phase error). At the beginning the ONN filter fails the recognition, but after 8 learning epochs the filter is able to recognize the edge as a vertical edge. Right: the backpropagation algorithm allows the recognition of the image for increasingly distorted input features. This backpropagation algorithm is suitable for implementing filter training in an ONN-CNN.

The remaining neural network layers are trained on the new dataset, achieving a recognition accuracy on the training set of 100% and on the test set of 95%. The original CNN, in comparison, reported better accuracy on the test set, of 97%. The reason for the worsening of the neural network performances is attributed to the cases in which the ONN fails the feature edge extraction. In fact, insufficient training of the ONN (just using HLR) also leads to recognition errors. The implementation of a backpropagation algorithm to the ONN layer would allow to increase the recognition performance in the network. We therefore implemented and tested a backpropagation scheme in our simulations. In Figure 10 we show an input image feature that should be recognized as a vertical edge. However, when the ONN is trained with the HLR the recognition fails. A cost function C = (φ_*train*_–φ_*out*_)^2^/2 is calculated from on the phases of the desired output φ_*train*_ and the obtained output φ_*out*_. Assuming an exponential dependence of the rising and falling edge of the relaxation oscillator waveforms, the derivative in time can be derived and an improved coupling matrix calculated. During subsequent epochs of this training the phase error is reduced. In the example shown in Figure 10, the feature is recognized after 8 epochs of training. While blurred features (allowing 40% gray scale) were only recognized with 30% probability using the untrained ONN, 100% of the features were recognized with the trained ONN. The extension of the backpropagation algorithm to the entire ONN-CNN is yet to be implemented, but is expected to boost the recognition performance. In addition, the direct implementation of the backpropagation algorithm would allow for direct training of a CNN algorithm on an ONN platform and should ultimately result in an increase of the training speed. Despite the reduction in recognition performances, the proposed ONN implementation allows for a reduction of the number of parameters that need to be trained by the network. In fact, 45 parameters undergo training for 5 CNN filters of 3 × 3 pixels size, however, only 36 parameters need to be trained for a single ONN that performs all filtering actions. The number of parameters to be trained is therefore reduced of 20%: this can represent an important advantage in terms of speed and power consumption when training larger networks. In addition, a further acceleration of the network speed and a further reduction of the number of memory accesses is achieved by the parallel processing of 5 filters from a single ONN unit, whilst in the standard CNN these five convolution actions are performed sequentially.

### Benchmark

In this section we benchmark the convolution operations conducted with the ONN compared to a conventional CPU or GPU. We assume that the first layer of the convolutional neural network presented in this paper is integrally realized via ONN filters operating in parallel. The first layer of the CNN consists of 64 filters of 3 × 3 dimension passing through a 27 × 27 pixel image with a stride of 2, accounting to total of 13 × 13 operations per filter. Assuming that each ONN can perform 5 filtering actions inherently, a total amount of 13 × 13 × 64/5 ≈ 2,200 ONNs is required, which corresponds roughly to 20,000 oscillator units and 80‘000 memristors for implementing the coupling. Assuming a minimum feature size of 100 nm for both the VO_2_ oscillators as well as the memristor, the total estimated area would be around 0.001 mm^2^.

For calculating the power consumption of the circuit, we refer to Shukla et al. (2016), Corti et al. (2020), that demonstrate operations of the oscillators at the power *P* = 20 μW with a scaled supply voltage <1 V and f = 3 MHz frequency operation. The total energy for the ONN to process one image with 64 filters at 3 MHz, including the waiting time of 5 oscillating period for the output stabilization, is calculated as

P×f×5=0.6⁢μ⁢J/f⁢r⁢a⁢m⁢e

Similarly, assuming the mean value of the coupling resistance to be around 100 kΩ, and the voltage drop across it 0.7 V, the total energy consumption of the memristors is calculated to be 3.4 μJ/frame.

Scaling of the device dimensions, it is envisioned that the VO_2_ oscillator could be driven with 1 μW @ 0.3 V at a moderately increased oscillation frequency of 20 MHz. Moreover, through improved processing and resulting device uniformity, the coupling strength could be weakened allowing 1 MΩ coupling resistance (Shukla et al., 2015). The figure of merit for such a scaled system would improve by 3 orders of magnitude resulting in an energy consumption of 3 nJ/frame.

For conducting the same operation, a standard GPU needs to perform (13 × 13) convolutions × 64 filters × (3 × 3) pixels/filter = 97,344 multiply-accumulation operation, that correspond to around 200,000 flops. In Intel’s CPU Core I9, which runs 1 TFLOP/s at 95 W, the total energy accounts for 20 μJ/frame; in the NVIDIA Tesla V100 GPU, that operates 120 TFLOP/s @ 300 W, the total energy is 500 nJ/frame (Table 2). We can conclude that the ONN system, when built with the current VO_2_ technology, is operating now at less power consumption of a conventional CPU, and given the scaling capabilities presented in other works, has the possibility of outperforming the top GPU available on the market. This analysis has been conducted not considering the peripheral circuitry that the ONN system will require, and therefore should be taken just as a projection of the potentiality of this technology and as an indication on the reduction in power consumption that this architecture can bring. Further benchmark should, however, be conducted at a stage when the technology is more advanced, to compare the performances to other specialized hardware that serve as accelerators for neural networks applications.

**TABLE 2:** Benchmark of the ONN technology against currently available platforms for convolutional neural network applications.

	**ONN (current)**	**ONN (projected)**	**CPU Intel’s Core I9**	**GPU Tesla V100**
Frames/s	0.6 × 10^6^	20 × 10^6^	5 × 10^6^	600 × 10^6^
Energy/frame	3.4 μJ	3 nJ	20 μJ	500 nJ
TFLOP/s	0.12	4	1	120
TFLOP/s W^–1^	0.06	67	0.01	0.4

## Conclusion

A concept for exploiting oscillatory neural networks as hardware accelerators in convolutional neural networks is presented in this paper. A 4-nodes oscillatory neural network was built with scaled VO_2_ oscillators’ technology on a Si platform. We show that the time-encoded output signal can store up to 5 trained filters and performs the equivalent function of multiple digital convolutional filters in a neural network. We expand the concept to a 3 × 3 VO_2_-ONN trained with Hebbian learning rule and simulate back-propagation for performance optimization. With the 3 × 3 filter and a transfer learning approach, we show that multiple digital filters of a CNN can be trained on a single ONN platform, achieving competitive recognition performances.

## Data Availability Statement

The raw data supporting the conclusions of this article will be made available by the authors, without undue reservation, to any qualified researcher.

## Author Contributions

EC and SK ideated and designed the concepts and experiments proposed in this work. EC was responsible for the device fabrication, the measurements, circuit simulations, and the neural network coding. JC conducted the characterization on the crossbar devices. KN and JR conducted the deposition and annealing of the VO_2_ films. KM, BG, and AI were involved in the discussion, experiment design, and editing of the manuscript and provided valuable input at multiple stages of this work. All authors contributed to the article and approved the submitted version.

## Conflict of Interest

EC, JC, KM, BG, and SK were employed by IBM. The remaining authors declare that the research was conducted in the absence of any commercial or financial relationships that could be construed as a potential conflict of interest.
